# Unique responses of the fixed stoichiometric TRPC1–TRPC5 concatemer to G proteins

**DOI:** 10.3389/fphys.2024.1392980

**Published:** 2024-09-27

**Authors:** Hana Kang, Insuk So

**Affiliations:** ^1^ Department of Physiology and Biomedical Sciences, Seoul National University College of Medicine, Seoul, Republic of Korea; ^2^ Institute of Human-Environment Interface Biology, Seoul National University, Seoul, Republic of Korea

**Keywords:** TRPC5, TRPC1, heteromer, homomer, concatemer, G protein, Englerin A, GTPγS

## Abstract

Transient receptor potential canonical (TRPC)5 channel is a non-selective cation channel that plays a significant role in membrane depolarization and calcium influx. TRPC5 not only forms homotetramers itself but also heterotetramers with TRPC1. However, accurately testing and confirming these heterotetrameric channels at specific ratios has proven challenging. Therefore, creating heteromeric concatemers of TRPC5 and TRPC1 with a fixed stoichiometry of 1:1 becomes essential. This study aims to meticulously identify and reaffirm the properties of TRPC5 homomers and heteromers with a 1:1 fixed stoichiometry to determine the optimal ratio for the TRPC1/5 heterotetramer. The overall characteristics were consistent with those of the previous studies, but several specific features were different. The TRPC1–TRPC5 concatemer is activated by Englerin A and G_i_QL, whereas carbachol alone does not trigger its activation. Additionally, G_q_QL significantly inhibited the current when co-expressed with the concatemer. Interestingly, carbachol can activate the TRPC1–TRPC5 concatemer in the presence of internal GTPγS, highlighting the influence of intracellular signaling conditions on its activation. Meanwhile, the TRPC5–TRPC5 concatemer is responsive to both carbachol and Englerin A. In conclusion, we provide evidence that the TRPC1–TRPC5 heteromeric concatemer with fixed stoichiometry need specific conditions to respond to carbachol, whereas the TRPC5–TRPC5 homomeric concatemer responds physiologically to carbachol. Additional research may be necessary to ascertain the optimal stoichiometry for the TRPC1–TRPC5 concatemer to enhance its electrophysiological properties.

## Introduction

Transient receptor potential canonical (TRPC) channels are calcium-permeable, non-selective cation channels that consist of seven members. Among the seven group members, TRPC1, TRPC4, and TRPC5 channels are classified as a subgroup that have similar stimulation processes (Schaefer et al., 2000; Kim et al., 2019a). The transient receptor potential canonical (TRPC)1 channel is widely distributed in mammalian cells. In mammalian cells, TRPC5 has only a single isoform, but it can form heteromeric channels with TRPC1 in the mammalian brain (Strubing et al., 2001). Subsequently, TRPC1 forms heterotetrameric channels with either TRPC4 (we use the term TRPC1/TRPC4 for heteromer) or TRPC5 (TRPC1/TRPC5) subunits and is involved in regulating calcium permeability and membrane potential of the plasma membrane (Zholos, 2014; Kim et al., 2019b). TRPC1/TRPC4 and TRPC1/TRPC5 have similar activation processes but slightly different desensitization (Myeong et al., 2018). The TRPC1/TRPC4 heteromer was desensitized via PIP_2_ depletion and Ca^2+^, whereas TRPC5/TRPC1 was desensitized via PIP_2_ depletion and PKC phosphorylation (Ko et al., 2019a).

Molecular mechanisms of tetramerization among TRPC4 and TRPC5 channels were addressed using the FRET method and size-exclusion chromatography (Schindl et al., 2008; Lepage et al., 2009). A part of first ankyrin repeat domain (ARD) at that time (69–98) was responsible for the homotetramerization process of TRPC5 (Schindl et al., 2008), and parts of the third and fourth ARD (87–172) and N-terminus coiled-coil domain (CCD) at that time (254–304) are important in the homotetramerization process of TRPC4 (Lepage et al., 2009). According to recent up-to-date domains (Duan et al., 2018; Vinayagam et al., 2018; Duan et al., 2019; Vinayagam et al., 2020; Wright et al., 2020; Song et al., 2021), they correspond to the second ARD (69–98) of TRPC5, the second and third ARD (87–172), and the helix–loop–helix (HLH) domain (254–304) of TRPC4. A recent study suggested similar results using the FRET method and thorough electrophysiology. With a number of N-terminal truncation mutants of TRPC4 channels, we suggested that 98–124 residues in the N-terminus (3rd ARD) and 700–728 residues in the C-terminus (connecting-helix) play a huge role in the homotetramerization process of TRPC4 (Myeong et al., 2014). Both N-terminal and C-terminal cytoplasmic domains seem to play a crucial role in the tetramerization process among TRPC4 and TRPC5. ARDs and the connecting helices (or rib helices) are of special interest (Kim et al., 2020).

The molecular mechanistic study for the heteromerization process is unfortunately limited despite its significance. One study so far represented specific domains responsible for the heteromerization process of TRPC1/4 and TRPC1/5 channels. Using the FRET method and electrophysiological recording with various truncation mutants, we suggested that 700–728 residues (connecting-helix) of TRPC4 and 707–735 residues (connecting-helix) of TRPC5 are important for heteromerization with TRPC1 (Myeong et al., 2016). However, it is surprising that TRPC1 utilizes different domains for TRPC4 and TRPC5. For TRPC1/4, the 725–745 region of TRPC1 is used as an inter-subunit interface, while the 673–725 region is used for TRPC1/5. Topologic analysis based on sequence alignment and the cryo-EM structure of TRPC4 and TRPC5 suggests that both regions of TRPC1 correspond to the putative connecting-helix of the TRPC1 channel. The N-terminal regions of TRPC1 (N-terminal CCD or HLH region: 188–278 residue) and the N-terminal regions of TRPC4 (N-terminal CCD or HLH region: 228–257 residue) or TRPC5 (N-terminal CCD or HLH region: 229–250 residue) were also involved in heteromerization (Myeong et al., 2016). Thus, ARD-HLH domains-connecting helix (rib helix)-C terminal CCD are involved in the heteromer formations. Since the cytosolic ARD embraces the CCD in the center and the connecting (or rib) helix contacts with ARD from the next subunit, our previous results match well with recent results of the TRPC4 (Duan et al., 2018; Vinayagam et al., 2018; Vinayagam et al., 2020) or TRPC5 structure (Duan et al., 2019; Wright et al., 2020; Song et al., 2021). Additionally, the cryo-EM studies have highlighted conserved features within the intracellular regions of TRPC channels. Notably, the pre-S1 elbow is located in the N-terminal domain, with the connecting helix running parallel to the membrane bilayer, underscoring a common architectural theme. A particularly intriguing aspect is the conservation of the binding interface with the Gα_i_ protein exclusively within the N-terminal ankyrin repeat domain of TRPC4 and TRPC5 channels. This specificity suggests that TRPC4 and TRPC5 may uniquely interact as direct modulators of Gα proteins within the TRP channel subfamily (Won et al., 2023). Furthermore, the conservation of the binding interface between the Gα protein and its effector molecules, as observed in the Gα_i_-bound TRPC5 cryo-EM structure, highlights a crucial aspect of signal transduction mechanisms involving these channels (Lyon et al., 2013; Won et al., 2023).

TRPC1 plays a tricky role in the channel field of the TRP1/4/5 subfamily. First, TRPC1 acts as a negative regulator for TRPC4/5. In neurodegenerative diseases like Huntington’s disease (HD) and Parkinson disease (PD), TRPC1 protects neuronal cell death by reducing the Ca^2+^ influx (Hong et al., 2015b). Following this hypothesis, a larger effect of TRPC1 knockout on cellular activity occurs in HD and PD than that of TRPC heteromer knockout. Interestingly, TRPC1 depletion induced double-rectifying I–V curves in synovial sarcoma cells (Muraki et al., 2017). Second, TRPC1 acts as a positive regulator for Na^+^ influx through TRPC channels to induce cell death in A498 and HS578T cells (Ludlow et al., 2017). Lastly, in many cases, TRPC1/4 and TRPC1/5 heteromers contribute to cell excitability by depolarizing the membrane potentials in neurons (Broker-Lai et al., 2017; Schwarz et al., 2019; Chu et al., 2020). It is rather surprising, however, that the effects of homomeric knockout (TRPC4 or TRPC5) and heteromeric double knockout (TRPC1/4 or TRPC1/5) are similar in terms of neuronal activity.

However, it has been hard to test and confirm the heterotetrameric channels with fixed ratios (Kim et al., 2014). Previous experiments were carried out by co-expressing TRPC5 and TRPC1 channels and determining the ratio by examining the shape of the current–voltage curve (I–V curve) or fluorescent density of the fluorescent protein tagged to TRPC channels. Certain fluorescent ratios seemed to form heteromers based on the previous studies (Myeong et al., 2018), but this also was not enough to authenticate the fixed ratio of TRPC1/5 heteromers as the structure of the heteromer has not been revealed. Thus, the heteromeric concatemers of TRPC5 and TRPC1 were made to get a fixed stoichiometry of 1:1 in order to find out the best stoichiometry of the TRPC1/4/5 tetraheteromer. We used the term TRPC1-5 concatemer for this construct.

Here, we started to test the TRPC1–5 concatemer. To provide a reasonable comparison, TRPC5–5 homotetrameric channels were tested along TRPC1–5. At the same day, we tested two types of concatemers for both the TRPC1–5 heteromer and TRPC5–TRPC5 homomer. The characteristics of TRPC1–5 and TRPC5–5 concatemeric channels were tested to confirm the regulation of the channels by the GPCR pathway and direct stimulation of the channel. Englerin A was tested at the end of each experiment for positive control to reconfirm that the concatemeric channels function properly. We also tested whether Gα_q_(Q209L) activation inhibits the activity of both TRPC1–5 and TRPC5–5. In addition, Gα_i2_(Q205L) was found to significantly activate the concatemeric channels with fixed stoichiometry. Furthermore, we investigated whether GTPγS could significantly increase the current in both TRPC5–5 and TRPC1–5.

## Methods

### Cell culture

Human embryonic kidney (HEK)293 cells were purchased from American Type Culture Collection (ATCC, VA). HEK293 cells that were stably expressing tetracycline-regulated human TRPC1–5 have been described previously (Zeng et al., 2004; Akbulut et al., 2015; Naylor et al., 2016). TRPC1–5 heteromeric concatemers were stably expressed in T-REx293 cells. All the cells were incubated in Dulbecco’s modified Eagle’s Medium (DMEM) supplemented with 10% heat-inactivated FBS, penicillin (100 U/mL), and streptomycin (100 μg/mL) at 37°C in a 5% CO_2_ humidified incubator. The modified HEK293 cells (T-REx293 cells) were supplemented with selection antibiotics blasticidin (5 μg/mL) and Zeocin (250 μg/mL) (Invitrogen) (Akbulut et al., 2015). To induce the expression of channels in T-REx293 cells, 1 μg/mL tetracycline was added to the media before it was seeded in a 12-well plate for whole-cell patch clamp recordings.

### Transfection of T-REx293 TRPC5–TRPC1 stable cells and HEK293 cells

Modified and stably expressing the tetracycline repressor HEK293 cells, T-REx293 cells were purchased from Invitrogen. T-Rex293 cells stably expressing human TRPC1–5 were maintained in the given medium above. An amount of 150 μL of 70%–80% confluent 100φ plate was seeded to 1 well/12 well each. TRPC1–5 stable cells were transfected at 60%–70% confluence with 0.5 μg/well of the pcDNA3 vector containing the cDNA for M receptor, GiQL, or GqQL mixed with 100 ng/well of pEYFP-N1 (Clontech) when no pEGFP was tagged. The transfection reagent TurboFect was added at a 1:2 ratio of DNA to the reagent, as detailed in the manufacturer’s protocol. After tetracycline inducement, current from TRPC1–5 was recorded.

TRPC5–5 homomeric concatemers and TRPC1–5 heteromeric concatemers were transfected to HEK293 cells using FuGENE 6 and TurboFect, respectively. Extra fluorescent protein was not added to TRPC5–5 homomeric concatemers as EGFP was tagged human TRPC5–TRPC5–EGFP cDNA. When using the FuGENE 6 transfection agent, a 1:3 ratio of DNA to the reagent was necessary for the ideal procedure. On the other hand, during TRPC1–5 transfection with TurboFect, pEYFP-N1 was also added. Co-expression of TRPC channels with G-proteins or receptors was achieved through a channel to G-protein transfection ratio of 1:1. After 24 h, the cells were trypsinized and transferred to a small recording chamber (RC-11, Warner Instruments) for whole-cell recording.

### Generation of TRPC1–TRPC5 and TRPC5–TRPC5 concatemers

**FIGURE 1:**
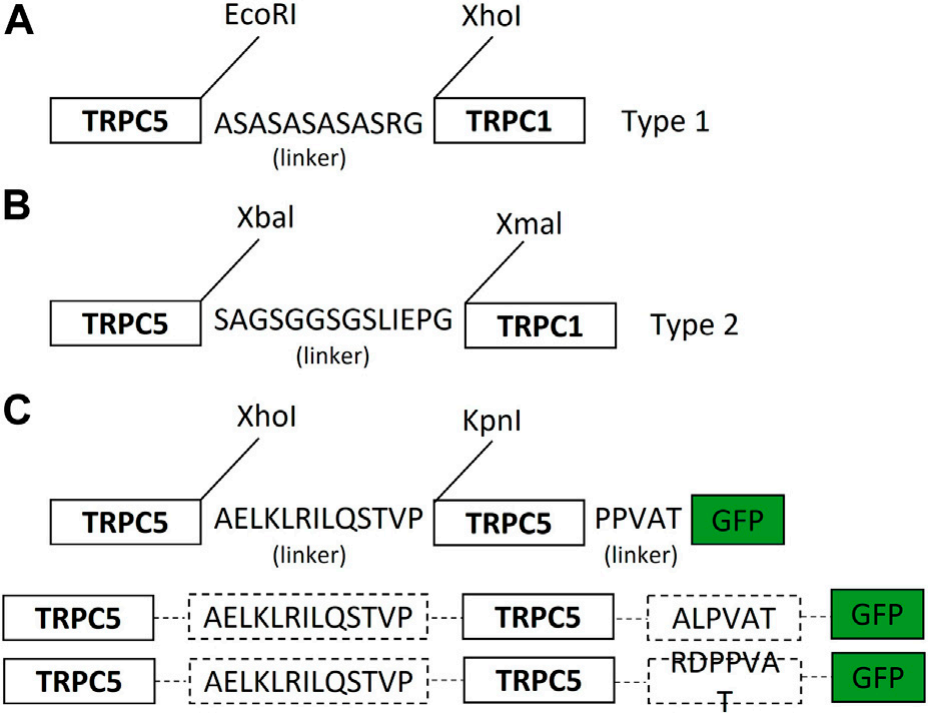
Schematic diagram of the TRPC1–TRPC5 concatemer and TRPC5–TRPC5 concatemer. **(A)** TRPC1–5 heteromer concatemer construct. Cloning of the TRPC1–5 concatemer was facilitated by flanking in the 10-amino acid linker (ASASASASAS) by the AgeI and SacII restriction sites using pcDNA™4/TO between the EcoRI and XhoI restriction sites using Gibson Assembly. (Further explanation in Ludlow et al., 2017). **(B)** Simple diagram of the TRPC1–5 concatemer. TRPC1–5 type 2 was generated via using non-cutter human TRPC5 and TRPC1 using Xbal and Xmal. Ten-amino acid linkers (GSGGSGSLIS) were flanked in between. SacI and SaII were used to insert the TRPC5–TRPC1 construct into the pUC57 vector. Based on the pUC57 vector, the TRPC5 gene was inserted into the head of the TRPC1 construct. This concatemer construct was placed on the mammalian expression vector. **(C)** TRPC5–TRPC5 concatemer construct was produced by flanking in AELKLRILQSTVP (L1A) linker and PPVAT linker using XhoI and KpnI with EGFP tagged by AgeI. Based on pEGFP-N1 vector, L1A was inserted in between human TRPC5.

The human TRPC1–5 concatemer cloned with a 10-amino acid linker (ASASASASAS) flanked by the AgeI and SacII restriction sites was introduced into pcDNA™4/TO between the EcoRI and XhoI restriction sites using Gibson Assembly^®^ (New England Biolabs) (forward oligonucleotide: 5′ CCA​CTA​GTC​CAG​TGT​GGT​GGA​ATT​CA CCG​GTG​CCA​GCG​CAT​CCG​CTT​CTG​CCT​CCG 3′; reverse oligonucleotide: 5′ GTT​TAA​ACG​GGC​CCT​CTA​GAC​TCG​AGC​CGC​GG GAT​GCG​GAG​GCA​GAA​GCG​GAT​GCG 3′). TRPC5, including an N-terminal Kozak sequence, was inserted upstream of the linker between the KpnI and AgeI restriction sites using hTRPC5/pcDNA™4/TO (Zeng et al., 2004; Rubaiy et al., 2017b) as a PCR template; forward primer: 5′GCT​GGT​ACC​GCC​ACC​ATG3′; reverse primer: 5′TGA​CAC​CGG​TGA​GGC​GAG​TT GTAACTGTTCTTC3′). TRPC1 was inserted downstream of the linker between the SacII and XbaI restriction sites (Figure 1). HEK293 cells stably expressing the TRPC1–5 construct were then generated for tetracycline-regulated expression, as for TRPC5 HEK293-Tet-cells (Zeng et al., 2004). For the hTRPC5–5 concatemer construct, two PCR products of hTRPC5, including two different restriction enzyme sites, were subcloned into the pEGFP-N1 vector using four (Nhe I, Xho I, Kpn I, and Age I) enzyme sites (New England Biolabs, United States). Among the amino acids of the linker between two TRPC5s, the leucine residue at the first position was changed to alanine for efficient expression (See Hong et al., 2015a, Figure 1D).

### Electrophysiology

The whole-cell patch-clamp was performed to measure the TRPC channel current in HEK293 cells. The transfected cells were trypsinized from the 12 wells and attached to coverslips in the small chamber on an inverted microscope (IX70, Olympus, Japan and TE 2000S, Nikon, Japan) for 7–10 min prior to patch recording. The currents were recorded using an Axopatch 200B amplifier (Axon Instruments). Patch pipettes were made from borosilicate glass and had a resistance of 3–5 MΩ when filled with normal intracellular solutions. The normal Tyrode(NT) contained 135 mM NaCl, 5 mM KCl, 2 mM CaCl_2_, 1 mM MgCl_2_, 10 mM glucose, and 10 mM HEPES, with the pH adjusted to 7.4 using NaOH. The internal solution contained 140 mM CsCl, 10 mM HEPES, 0.2 mM Tris-guanosine 5′-triphosphate, 0.5 mM EGTA, and 3 mM Mg-adenosine 5′-triphosphate, with the pH adjusted to 7.4 with CsOH. A voltage ramp pulse from +100 mV to −120 mV was applied for 500 ms at a −60 mV holding potential. Experiments were performed at room temperature (19°C–26°C). The recording chamber was continuously perfused at a flow rate of 1–2 mL/min. pCLAMP software (version 10.2) and Digidata 1440A (Axon Instruments) were used for data acquisition and application of command pulses. Data were filtered at 5 kHz and displayed on a computer monitor. Data were analyzed using pCLAMP (version 10.7) and Origin software (Microcal Origin, version 8).

### Perforated whole-cell patch-clamp

The whole-cell patch-clamp was performed with nystatin for perforated patch-clamp. A stock solution of nystatin was prepared at a concentration of 100 mg/mL in DMSO on the day of the recording. This stock solution was covered by aluminum foil to avoid light. The DMSO concentration was 0.2%. No filter was added before use. The patch pipette was dipped into the antibiotic-free solution for 2 s before filling with the nystatin solution to avoid impairment of the initial GΩ seal formation. The remainder of the pipette was back-filled with the internal pipette solution containing nystatin. The pipette potential was held at −60 mV. As the access resistance (Ra) decreased and electrical contact with the cell improved, a current transient due to the cell membrane capacitance (Cm) was observed. Recordings could commence once the Ra stabilized at a suitable value and then was compensated for. Stable Ra <30 MΩ by 30 min was obtained (Jeon et al., 2022). Other methods were redundant with the Electrophysiology section.

### Confocal imaging

Confocal imaging HEK293-T cells were cultured on Poly-L-lysine-coated 18-mm glass coverslips for confocal image acquisition. The cells were transfected with 2 μg of either ECFP-tagged hTRPC5 or ECFP-tagged hTRPC5-hTRPC1cDNA with or without fluorescence protein-untagged hTRPC5 with EYFP-PH using PEI (Polysciences; MW 4000, 1 mg/mL) in a 1:4 ratio of total DNA (μg) to PEI (μL). High-resolution confocal images of HEK293-T cells were acquired on a Zeiss LSM 980 microscope with an Airyscan detector using a ×63, 1.4 NA oil immersion Plan-Apochromat objective. A zoom factor of ×2 and a frame size of 1,713 × 1,713 were used for all images, resulting in an XY pixel size of 38.7 nm. A laser power of 1% and ∼2% was used for the 445-nm and 514-nm lasers, respectively. The emission wavelengths of the respective fluorescence were detected at 475 nm and 524 nm. The cells transfected with hTRPC5–hTRPC5–EGFP with or without hTRPC5–hTRPC1–ECFP were stained with WGA (5 μg/mL, Invitrogen) for labeling the plasma membrane before imaging. All settings were the same with TRPC5–5 confocal imaging, except that TRPC5–5 was taken by a 5% 488-nm laser of Em 509, and WGA was used for the 0.5% 590-nm laser of Em 618.

### Solutions and drugs

For all TRPC channel recordings, a physiological salt solution and Normal Tyrode solution were employed. The Normal Tyrode solution contained 135 mM NaCl, 5 mM KCl, 2 mM CaCl_2_, 1 mM MgCl_2_, 10 mM glucose, and 10 mM HEPES, with the pH adjusted to 7.4 using NaOH. The Cs-rich external solution contained 140 mM CsCl, 2 mM CaCl_2_, 1 mM MgCl_2_, 10 mM glucose, and 10 mM HEPES, with a pH of 7.4 adjusted with CsOH. The pipette solution for whole-cell recording contained 140 mM CsCl, 0.5 mM EGTA, 10 mM HEPES, 3 mM Mg-ATP, and 0.2 mM Tris-GTP, with a pH of 7.3 adjusted with CsOH. Toxin was purchased from Calbiochem (La Jolla, CA), and carbachol, HEPES, and GTPγS were purchased from Sigma. The 1ul stock solution of 100 mg/mL Nystatin in DMSO was added to the 140 mM KCl, 10 mM HEPES, and 10 mM EGTA with a pH of 7.2 with the pipette solution. They were all purchased from Sigma.

## Results

### Ion permeability and I–V curve of TRPC1–TRPC5 heteromeric concatemers

**FIGURE 2:**
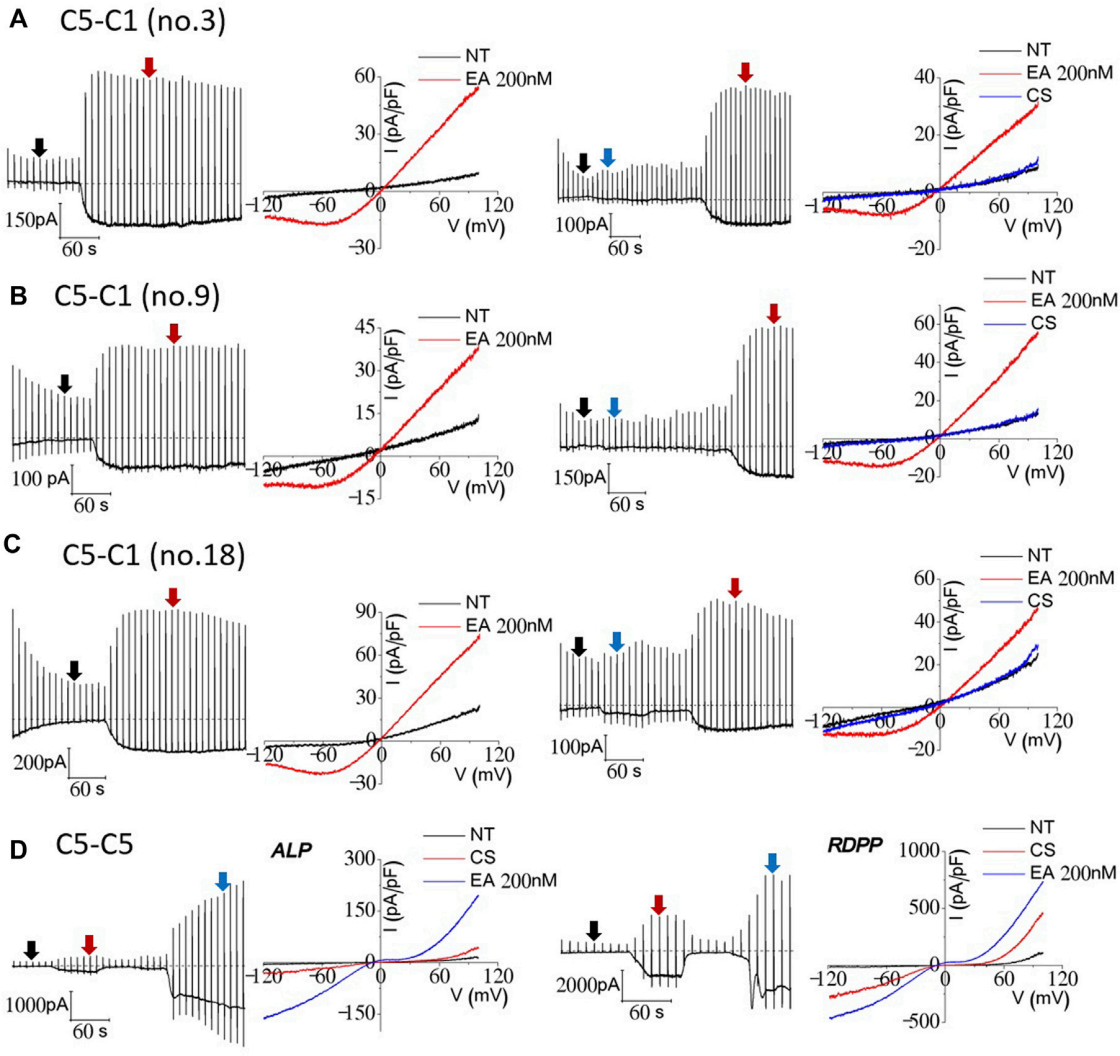
Cesium- and Englerin A-induced currents of TRPC1–TRPC5 and TRPC5–TRPC5 concatemers. A total of 14 cell lines were tested prior to TRPC1–5 heteromer stable cell usage (**A–C**). The three cell lines with the biggest current inducements were no. 3, no. 9, and no. 18. All three of the cell lines were tested with EA 200 nM at first to confirm the heteromeric current. After that, the external solution was changed from NT to a cesium-rich solution for recording the current. The currents were recorded in TRPC1–5 stably expressing tetracycline-induced HEK293 cells using the wholecell patch-clamp technique. Tetracycline inducement was carried out 24 h before the patch-clamp recording. **(D)** HEK293 cells transiently transfected with EGFP-tagged TRPC5–5 concatemers were used for whole-cell patch-clamp recording. Cells with similar brightness of EGFP were patched prior to recording. At the holding potential of −60 mV, the ramp pulse was applied from 100 mV to −120 mV at every 20 s. The I–V curve of the current was recorded at the peak each time.

To investigate the electrophysiological properties of TRPC1–5 heteromeric concatemers, we initially transiently transfected, but we failed to obtain the typical current from the concatemer from most of the experiments. Thus, we made stable cell lines and screened the colonies. Stable HEK293 cell lines inducibly expressing a TRPC1–5 concatemer were established. For screening, we used Englerin A as an agonist (Jeong et al., 2019). We found 14 stable cell lines showing typical I–V curves of TRPC1/5 heteromers. Among the 14 stable cell lines tested, three stable cell lines, no. 3, no. 9, and no. 18, were selected and used for the following experiments (Figures 2A–C). To provide a reasonable comparison, TRPC5–5 homotetrameric channels were tested along with TRPC1–5. On the same day, we tested two types of concatemers for both the TRPC1–5 heteromer and TRPC5–5 homomer. The TRPC1–5 concatemer had an outwardly rectifying shape of the I–V curve, whereas the TRPC5–5 concatemer had a double rectifying shape, which is very typical for heteromers and homomers. TRPC5–5 concatemers had significantly bigger current sizes (Figure 2). Measurement of the changes in the extracellular cesium concentration revealed that cesium increased the current even in the TRPC5–5 concatemer (Figure 2D). On the contrary, cesium did not increase the current in TRPC1–5 heterotetramers. In our previous study, we demonstrated that TRPC1–5 heterotetramers establish a pronounced pore field, leading to a reduction in the relative conductance of monovalent ions, specifically cesium (Kim et al., 2012). This suggests that the heteromeric assembly of TRPC5 and TRPC1 has inherent limitations in its pore opening capabilities, akin to those observed when TRPC5 and TRPC1 are co-expressed. Consequently, this implies that cesium ions alone are insufficient to enhance the pore permeability of these heteromers.

### TRPC1–TRPC5 concatemer was not activated by M receptor stimulation

**FIGURE 3:**
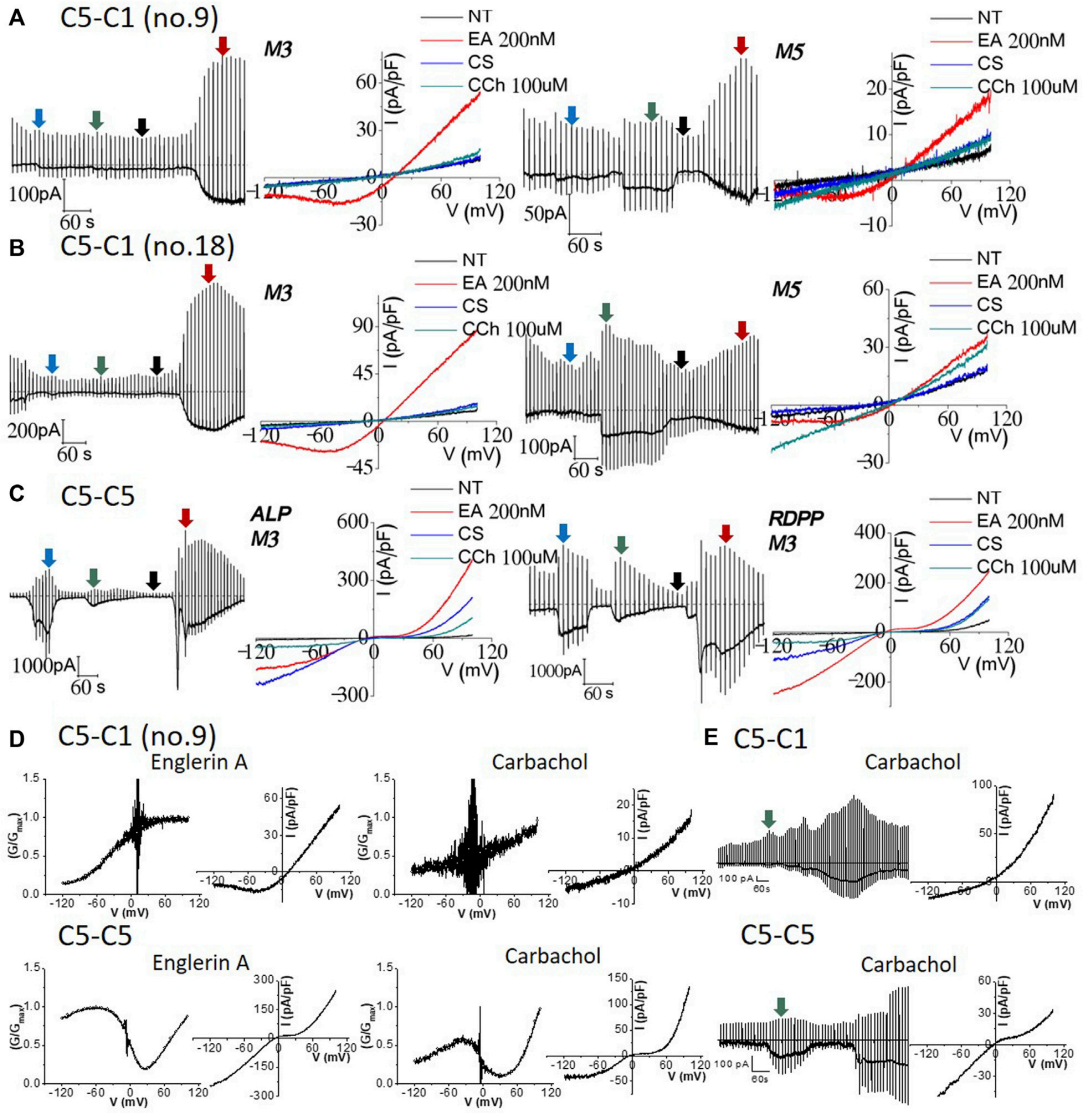
Effect of 100 μM carbachol on TRPC1–TRPC5 and TRPC5–TRPC5 muscarinic receptor-expressed cells. Full traces and I/V curves of heterotetrameric **(A)** TRPC1–5 no. 9 and **(B)** TRPC1–5 no. 18 following external solution change to the cesium-rich solution, 100 μM carbachol, and then 200 nM Englerin A stimulation. M3 receptor-expressing (**(A,B)** left) cells show good positive control with Englerin A activation but no carbachol stimulation at all. M5 receptor-expressing (**(A,B)** right) cells had inward specific activation but did not have the heteromeric current. Smaller Englerin A stimulation was observed as the cells were affected by carbachol stimulation. **(C)** Cells expressing the TRPC5–5 and M3 receptor were exposed to cesium external solution change and carbachol stimulation, followed by Englerin A activation. Englerin A activation was the largest, cesium current increase was the second, and carbachol stimulation led to the smallest current inducement. **(D)** Characteristics of TRPC1–5 and TRPC5–5 concatemer current. TRPC5–5 shows a double rectifying current–voltage (I–V) curve with diminished conductance. All conductance–voltage (G–V) were normalized from 0 to 1, with 1 being the maximum conductance; G/Gmax refers to normalized conductance. Its G–-V curve features a dynamic N-shaped curve, which is distinct from that of the TRPC1–5 heteromer. The TRPC1–5 heteromer shows an outward rectifying I–V curve. The G–V curve features a dynamic sigmoid-shaped curve. **(E)** Nystatin perforated patch mode with the preserved intracellular environment and Ca^2+^ signaling pathway resulted in similar result with whole-cell patch-clamp. Raw trace represents the perforated patch-clamp induced by carbachol using Englerin A as the positive control (TRPC1–5 *n* = 3, TRPC5–5 *n* = 3). The I–V curve was derived from the green segment of the full trace, representing the peak response to the external carbachol solution.

Since we recorded typical currents from TRPC1–5 concatemers, we investigated whether muscarinic stimulation induces currents in TRPC1–5 concatemers. Both cells expressing the TRPC1–5 concatemer, the transiently and stably expressing cells, did not show any significant response to M3 stimulation. On the other hand, inward current simultaneously increased, while the outward current remained constant for M5 stimulation (Figures 3A, B, right). However, the I–V curve of carbachol stimulation did not seem consistent to the previous studies. We observed linear I–V curve carbachol stimulation with the expression of the M5 receptor, which suggested that the expression of the M5 receptor induces some changes on other unknown structures rather than the TRPC1–5 structure itself (Figure 3B). Additionally, perforated whole-cell patch-clamp was performed to test the M receptor stimulation with carbachol in the preserved intracellular environment of the Ca^2+^ signaling pathway. Similar results as those of the whole-cell patch-clamp were observed (Figure 3E). To confirm the carbachol activity, the effect of M3 stimulation on the TRPC5–5 concatemer has been tested at the same time. Both the ALP and RDPP homomeric concatemers had an ideal I–V curve, that is, a double rectifying shape (Figure 3C).

### Inactivation of the TRPC1–TRPC5 concatemer after Gɑ_q_(Q209L) activation

**FIGURE 4:**
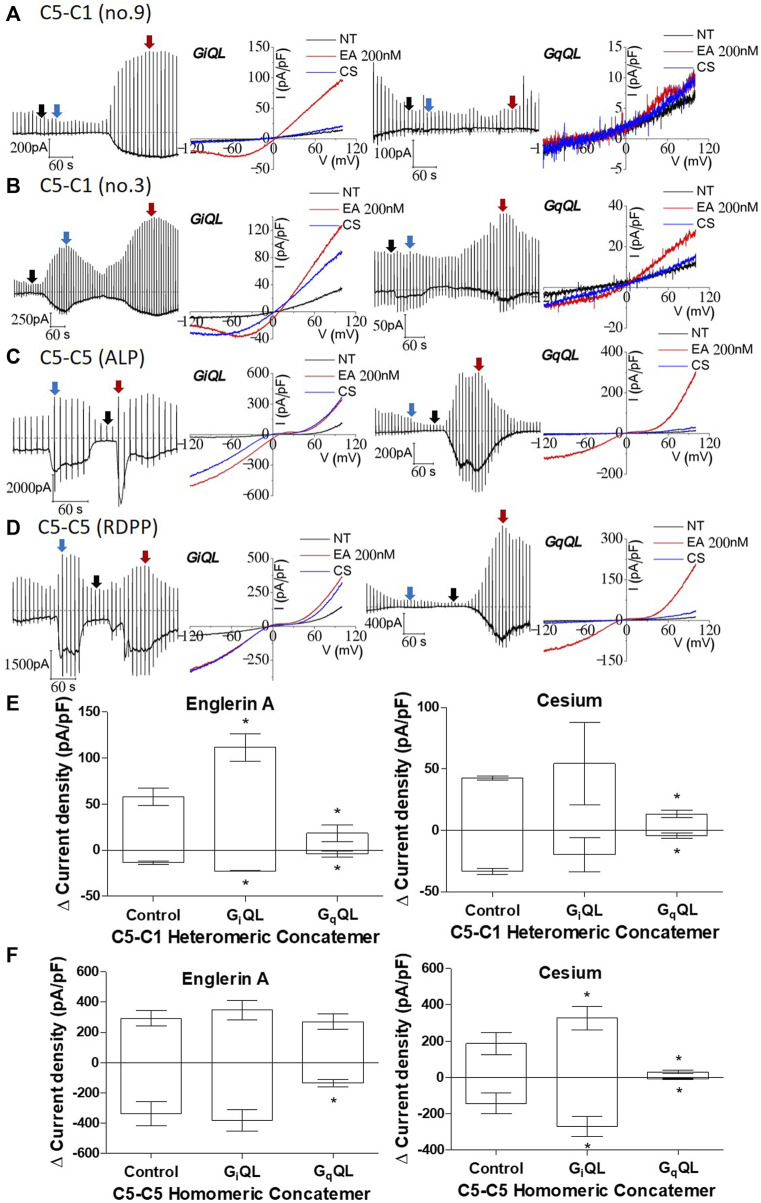
Inhibition of TRPC1–TRPC5 and TRPC5–TRPC5 by Gɑ_q_(Q209L) and activation of TRPC1–TRPC5 and TRPC5–TRPC5 by Gɑ_i2_(Q205L). [**(A, B)** right] TRPC1–5 concatemer co-expressed with Gɑ_q_(Q209L) was stimulated by 200 nM Englerin A at +100 mV. When the external solution was changed to the cesium-rich solution, no or small change in the current was observed. There was no Englerin A stimulation observed. [**(A, B)** left] TRPC1–5 concatemer co-expressed with Gɑ_i2_(Q205L) was stimulated with the same protocol and showed a significant increase. The color of the arrow, the peak or base of each stimulation, corresponds to the I–V curve color. **(C, D)** TRPC5–5 concatemer co-expressed with Gɑ_i2_(Q205L) (left) was stimulated with the same protocol and showed a significant increase on cesium exposure, but no changes were seen on Englerin A stimulation. In case of Gɑ_q_(Q209L) (right), the current decreased both on Englerin A stimulation or cesium exposure. **(E, F)** Summarized current density at −100 mV and +100 mV of TRPC1–5 and TRPC5–5 stimulated by 200 nM Englerin A and external high cesium concentration. Englerin A as a positive control (For TRPC5–5: control *n* = 7, G_i_QL *n* = 9, G_q_QL *n* = 6; TRPC1–5: control *n* = 7, G_i_QL *n* = 2, G_q_QL *n* = 2, *p-value < 0.05).

Next, we investigated whether G_q_, the downstream target of muscarinic stimulation, activates or inhibits the TRPC1–5 concatemer. In our previous study, we showed that G_q_ predominantly activated the TRPC1/5 heteromer when TRPC5 and TRPC1 were co-expressed (Myeong et al., 2018). Gɑ_q_(Q209L) is a constitutively active Q209L mutant of Gɑ_q_, which lacks the intrinsic GTPase activity. This means it exists in the GTP-bound active conformation. The expression of the active mutant Gɑ_q_(Q209L) is known to activate PLCβ that depletes PIP_2_ (Jeon et al., 2012; Kim et al., 2012; Myeong et al., 2018; Ko et al., 2019b). When the Gα_q_(Q209L) mutant is expressed alongside the TRPC1–5 concatemer, most cell lines do not exhibit activation, including that by Englerin A. Activation by Englerin A, when observed, was suppressed by PIP_2_ depletion in these Gα_q_QL-expressed TRPC1–5 concatemers (Figures 4A, B right, E). No current change or activation was observed with external cesium gradients in the TRPC1–5 concatemer. A similar result was observed in the TRPC5–5 concatemer (Figure 4C right, F). Interestingly, fast activation–deactivation was observed when activated via Englerin A at the TRPC5–5 concatemer expressing Gɑ_q_(Q209L) (Figures 4D, F). In cells expressing TRPC5–5 with Gα_q_(Q209L), Englerin A induced a smaller current compared to the control, yet this increase remains significant relative to the baseline inactive current (Figure 4F).

#### Gɑ_i2_(Q205L) increased the current in the TRPC1–TRPC5 concatemer

We investigated the effect of Gɑ_i2_, which is another downstream target of muscarinic stimulation. The Gɑ_i2_ isoform is known to be the most effective activator among the Gɑ_i_ subunits of TRPC4 (Jeon et al., 2012). TRPC5 has a comparable structure to TRPC4 and similar response to Gɑ_i2_(Q205L) (Jeon et al., 2012; Thakur et al., 2016). The no. 9 stable cell line expressing the TRPC1–5 concatemer had enhanced current that increased due to Englerin A stimulation but did not have cesium current increase. On the other hand, the no. 3 stable cell line expressing the TRPC1–5 concatemer rarely showed enhanced current due to cesium (Figures 4B, E). None of the previous studies regarding heteromer current showed such significant increase due to cesium. Most of the studies showed an increase in the cesium current for transiently transfected heteromer; TRPC1/4 had a homomeric I–V curve when TRPC4 and TRPC1 were co-expressed. This suggests that heteromers with fixed stoichiometry do not generally exhibit a notable increase in the cesium current. However, Englerin A-induced activation in the TRPC1–5 concatemer co-expressed with Gα_i2_(Q205L) may lead to maximized currents, indicating that G proteins can directly modulate the heteromer under ideal experimental conditions (Figure 4E).

In the TRPC5–5 concatemer co-expressed with Gɑ_i2_(Q205L), the response to cesium was maximized compared to any other stimulation (Figures 4C, D, F). The current increase reached close to that of Englerin A stimulation, which is known as the strongest agonist of TRPC5. Englerin A did not further increase currents from TRPC5–5 homomeric concatemer channels. All three types—control, G_i_QL, and G_q_QL—responded to Englerin A stimulation, verifying the reliability of the experimental results across different co-expressions.

### Internal GTPγS facilitated the response to carbachol in the TRPC1–TRPC5 concatemer

**FIGURE 5:**
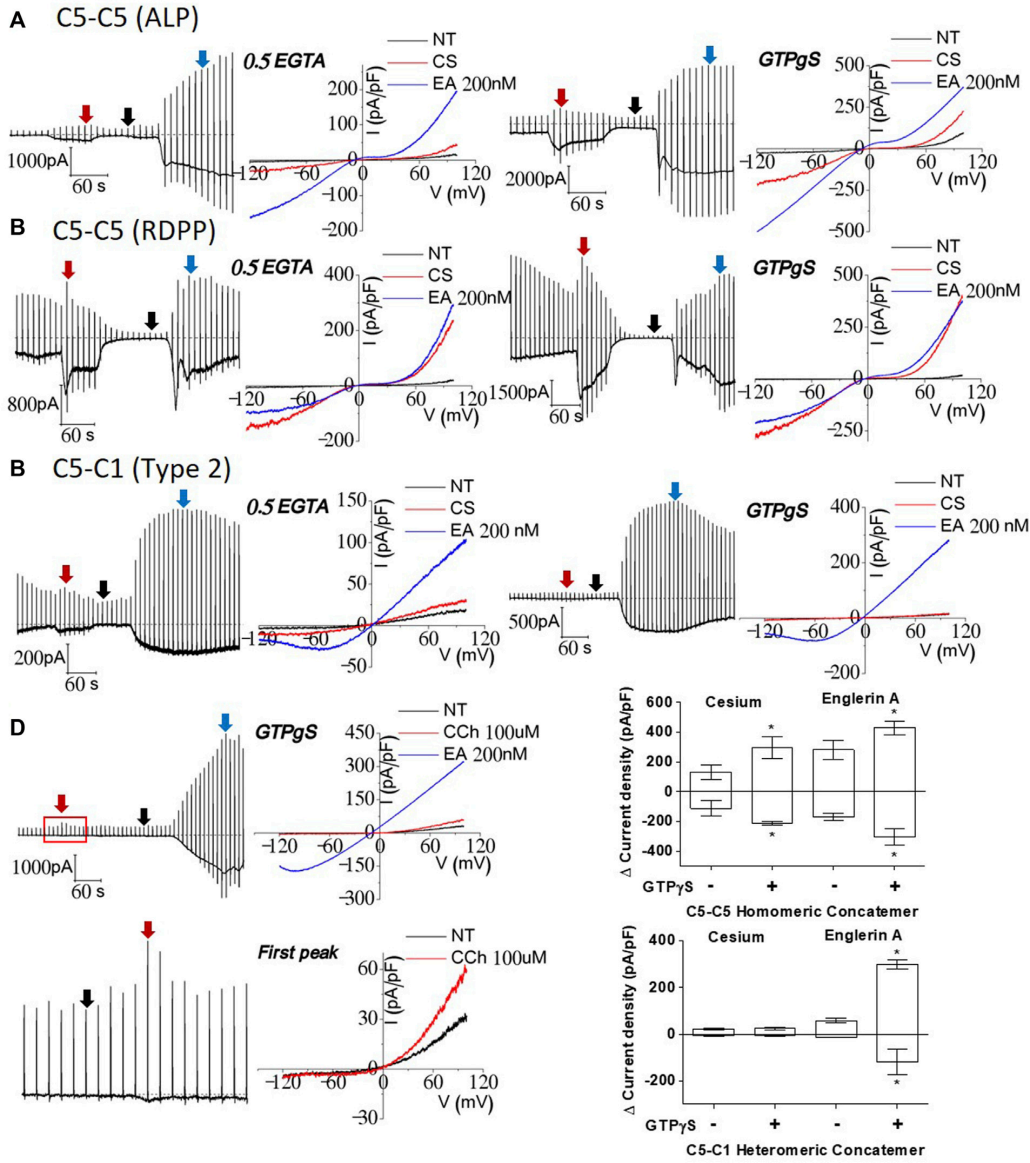
Effects of GTPγS on TRPC1–TRPC5 and TRPC5–TRPC5 concatemers; **(A, B)** TRPC5–5 homomer comparison of intracellular GTPγS and control internal solution. Both TRPC5–5 homomeric currents increased approximately twice with internal GTPγS in both the external cesium solution and 200 nM Englerin A stimulation. **(C)** TRPC1–5 heteromer comparison showed significant increase in 200 nM Englerin A stimulation. There was no current change in cesium-rich external solution. **(D)** Upon stimulation with 100 μM carbachol, current activity was slightly enhanced. The I–V curve showed heteromeric current. **(E)** GTPγS effect led to the max current, regardless of the kind of current enhancement. TRPC1–5 pore cannot be loosened in a cesium-rich solution by affecting G protein permeability (cesium *n* = 5, Englerin A *n* = 4, *p-value < 0.05).

Finally, we investigated whether GTPγS, a universal activator for TRPC channels, facilitates the response to carbachol in the TRPC1–5 concatemer. It is known that activation of G protein can stimulate the TRPC5/5 homomer, while TRPC1/5 has not been studied specifically (Schaefer et al., 2000). In the TRPC5–5 homomeric concatemer, GTPγS induced an increase in the basal current recorded instantly after cell rupture. The TRPC5–5 concatemer current increased after external solution change (NT to Cs) (Figures 5A, B). Additionally, the Englerin A effect was also enhanced as GTPγS already acts as an activator intracellularly. TRPC5–5 homomeric currents activated by Englerin A stimulation were maximal compared to any other stimulation for activation as well as the control (Figures 2–4). The external cesium-rich solution in the TRPC5–5 homomer with GTPγS could reach the amplitude similar to that of the Englerin A current (Figure 5B, right).

HEK293 cells are reported to endogenously express functional M3 muscarinic receptors (Zhu et al., 1998; Ancellin et al., 1999; Hakak et al., 2003; Luo et al., 2008; Capilupi et al., 2020). There was a slight current increase right after 100 μM carbachol stimulation, and then, it constantly decreased to basal levels after it reached the peak. Current increase by carbachol disappears without NT wash. To recheck if the peak of carbachol stimulation was successful, the IV curve at the peak was obtained, which seemed to be an ideal outward rectifying shape. When GTPγS and carbachol react internally and externally at the same time, the synergistic effects of all G protein pathways for activation might be observed. An amount of 200 nM of Englerin A was always used as a positive control at the end of the patch-clamp. Interestingly, the TRPC1–5 heteromeric concatemer had a similar I–V curve to that of 200 nM Englerin A after being stimulated with carbachol, but the response was delayed to reach the peak (Figure 5D). In the TRPC1–5 heteromer, enhanced Englerin A activation could be seen as well (Figures 5C, E).

**FIGURE 6:**
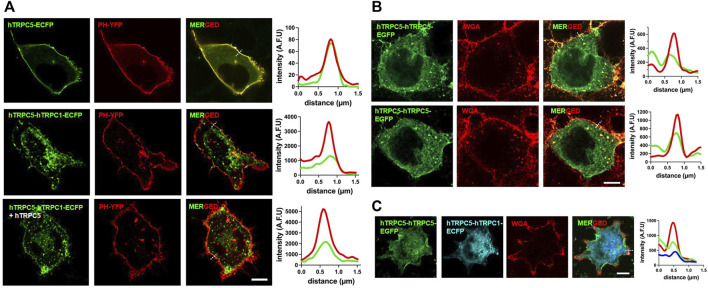
Localization of TRPC1–TRPC5 and TRPC5–TRPC5 concatemers. **(A)** Representative images of HEK293T cell expressing hTRPC5–ECFP or hTRPC5–hTRPC1–ECFP with or without hTRPC5 and with YFP-PH. The dashed line is used for line scan analysis. **(B)** Representative images of HEK293T cells expressing hTRPC5–hTRPC5–EGFP and stained with WGA for labeling the plasma membrane. The dashed line is used for line scan analysis. **(C)** Representative images of HEK293T cells expressing hTRPC5-hTRPC5-EGFP with hTRPC5-hTRPC1-ECFP and stained with WGA for labeling the plasma membrane. The dashed line is used for line scan analysis; Scale bars: 10 μm.

When the distribution of TRPC1–5 was examined with a confocal microscope or fluorescent microscope, TRPC1–5 was predominantly localized to the vesicles, unlike the TRPC5 homomer, which was primarily found at the plasma membrane. When the TRPC5 homomer was co-expressed with the TRPC1–5 heteromer, TRPC1–5 channels were located at the plasma membrane. The proportion, however, was very different compared to the homomeric TRPC5 (Figure 6). The most important observation was that both the TRPC5 homomer and the TRPC1–5 heteromer were observed at the plasma membrane when co-expressed. In addition, Western blot analysis and surface biotinylation reveal that the overall expression level of the TRPC1–5 concatemer is lower compared to that of the TRPC5–5 concatemer (data not shown). However, when adjusted for total protein amounts, the expression levels at the plasma membrane are comparable. Notably, co-expression with monomeric TRPC5 or the TRPC5–5 concatemer enhances the level of expression at the plasma membrane.

## Discussion

In the present study, we showed that 1) muscarinic stimulation did not induce any current in TRPC1–5 heteromeric concatemer, 2) GαiQL activates and GαqQL inhibits both TRPC1–5 heteromeric and TRPC5–5 homomeric concatemers, 3) EA induced a large current in TRPC1–5 heteromeric concatemer, and 4) muscarinic stimulation can induce a small current with outward rectifying I–V curve in TRPC1–5 concatemer when GTPγS was included in the pipette.

TRPC1 is often regarded as a negative regulator for TRPC4/5 (Hong et al., 2015b; Kim et al., 2019a). In neurodegenerative diseases like Huntington’s disease and Parkinson’s disease, TRPC1 protects neuronal cell death by reducing the Ca^2+^ influx (Hong et al., 2015b). In synovial sarcoma cells, TRPC1 depletion induced a double-rectifying I-V curve. We predict that the deletion of TRPC1 will exert a more profound impact on cellular activity compared to knockouts of TRPC heteromers, especially in neurological conditions such as Huntington’s disease and Parkinson’s disease. We do not claim that TRPC1 solely acts as a negative regulator. TRPC1/4 and TRPC1/5 heteromers frequently amplify cell excitability by depolarizing neuronal membrane potentials. Notably, the neuronal activity effects from knocking out homomeric (TRPC4 or TRPC5) or heteromeric (TRPC1/4 or TRPC1/5) channels are strikingly similar. On the other hand, TRPC1 was suggested to act as a positive feedback for intracellular Na^+^ ions (Ludlow et al., 2017). In A498 and HS578T cells, TRPC1 acts as a positive regulator for Na^+^ influx through TRPC channels to induce cell death. The TRPC research community has greatly enhanced our understanding of TRPC4/5 homomer channels through the identification and analysis of interacting proteins. Continuing studies into these interactions are expected to advance our comprehension of TRPC1’s multifaceted regulatory roles in TRPC4/5 channels, clarifying whether it acts predominantly as a negative or positive regulator. In addition, TRPC1 also acts as a negative regulator for the L-type Ca^2+^ channel or TRPV6. TRPC1 is also well-known to make a channel with STIM and/or ORAI, which is called a store-operated channel (SOC). Considering structures based on Cryo-EM data, it seems impossible for TRPC1 to form a heteromeric complex with TRPV6 or STIM and ORAI.

**FIGURE 7:**
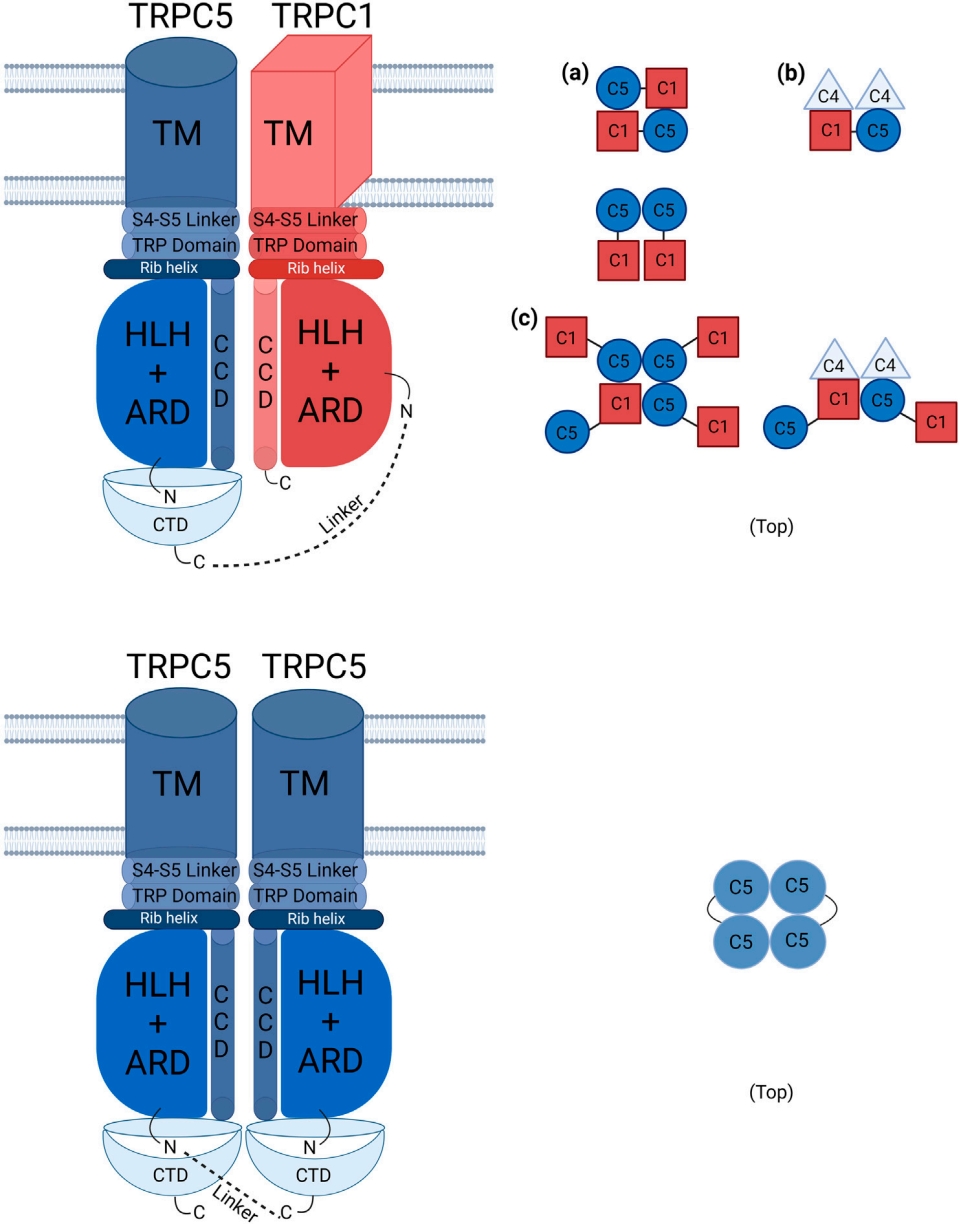
Schematic presentation of TRPC1–TRPC5 and TRPC5–TRPC5 concatemer structures with other possible combinations. The schematic illustration of TRPC channels shows defined stoichiometries for TRPC1–5 and TRPC5–5 connected by linker lines. The TRPC1–5 concatemer can form tetramers with TRPC1 in either cis or trans configurations. In stable cell lines, TRPC1–5 may form functional heteromer channels in a 1:3 ratio with endogenous TRPC5, which are more frequently activated by Englerin A compared to those in transiently transfected cells. **(a)** represents the most possible structure of TRPC1-5 concatemer that is 1:1. **(b)** may be the ideal structure that leads the stable cell lines of TRPC1–5 to show heteromeric characteristics as one TRPC1 forms the tetramer. Endogenous TRPC4 could influence the formation of the heteromeric channels. **(c)** shows the possible structure of oligomer (see also Lansky et al., 2023a). This structure may be the least abundant of all. TM: transmembrane domain, ARD: ankyrin repeated domain, CCD: coiled coil domain, CTD: C terminal domain, HLH: helix–loop–helix domain.

One big question from this study was why the TRPC1–5 concatemers did not react to the carbachol even with M3 or M5 receptor expression (Figure 3). The TRPC1–5 concatemer was activated by Englerin A and showed the typical outward rectifying I–V curve (Figure 2). Interestingly, the TRPC1–5 concatemer expressed with M5 showed an inward current increase to carbachol, but the I–V curve does not seem ideal to be confirmed as a heteromer current (Figure 3). After getting this result, we re-evaluated the previous results from other research groups (Rubaiy et al., 2017b; Ludlow et al., 2017). There was no carbachol experiment done with the specific TRPC1–5 concatemer. In the TRPC1–4 concatemer, SIP induced a smaller and less robust response than Englerin A (Rubaiy et al., 2017a; Jeon et al., 2021). However, Gd^3+^ did not activate TRPC1–4 concatemer channels (Rubaiy et al., 2017a). The answer to this question is still unknown and has not been figured out. Muscarinic stimulation activates TRPC4 and TRPC5 channels by two mechanisms, PIP_2_ depletion by DAG and direct binding of G protein. Considering the recent TRPC5-G_i3_ complex structure, the ARD may be the reason for the failure of the carbachol reaction. ARD plays a significant role in the G protein signaling pathway. Calmodulin binding to the rib helix of TRPC4 restricts the channel’s conformational flexibility, potentially limiting channel activation, as this constraint imposed by the ARD may influence the activation process. Additionally, it is notable that the TRPC1 structure lacks the region, following the CCD, a feature that is present in the TRPC5 structure, indicating structural variations that could influence the function (Vinayagam et al., 2020; Won et al., 2023). As shown in Figure 7, the homotetramer is able to fully function with a short linker. On the other hand, in the heterotetramer, TRPC1 might require a longer linker with TRPC5 (Figure 1). The short linker in TRPC1–5 concatemer constructs may result in the malfunctioning of the ARD.

Another possibility is that the activation mechanism of Englerin A is different from that of carbachol. Even in the C553/C558 mutant of TRPC5, the same result was obtained. The C553/C558 mutant was activated by Englerin A (Duan et al., 2018; Vinayagam et al., 2018; Jeong et al., 2019) but not by carbachol or Gα_i_(QL) (Hong et al., 2015b). Previous studies showed that Gɑ_q_(Q209L) completely inhibited TRPC5 when activated with agonist, carbachol, or Gα_i_(QL) (Jeon et al., 2012). However, when the homomeric concatemers were tested with cesium or Englerin A, cesium did not increase current, but Englerin A was able to activate the channel under the condition of Gɑ_q_(Q209L). The Englerin A-induced current decreased with fast deactivation, resulting in V shaped activation–deactivation (Figure 4). We showed that Englerin A binds to the inter-subunit interface and increases the current (Jeong et al., 2019; see also Song et al., 2021; Vinayagam et al., 2020; Wright et al., 2020). Independent of muscarinic receptor stimulation via ARD, Englerin A might increase the current by acting on the pore domain itself, including the selectivity filter.

Interestingly, in the TRPC1–5 concatemer, carbachol elicited the typical current response when applied extracellularly along with intracellular GTPγS. This suggests that G proteins other than Gα_i_ may play a role in regulating the response of the TRPC1–5 concatemer to carbachol (Kim et al., 2012; Wie et al., 2015a; Wie et al., 2015b). TRPC5–5 homomer G–V curve featured a dynamic N-shaped curve, distinct from that of the TRPC1–5 heteromer, as expected during perforated patch-clamp, which confirmed the characteristics of the heteromer under the condition of the preserved intracellular environment and Ca^2+^ signaling pathways (Park et al., 2023).

**FIGURE 8:**
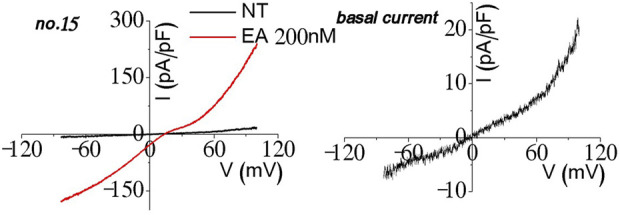
Homomeric current observation in the process of the TRPC1–TRPC5 heteromer stable cell line experiment.

During stable cell generation and testing, few of the cell lines showed homomeric current when activated (Figure 8). Even though the cell lines expressing the TRPC1–5 concatemer were selected with Zeocin and blasticidin (*n* = 30), about half of them did not activate (*n* = 15), one-third (*n* = 10) of the cell lines did not activate enough to test characteristics other than Englerin A stimulation, and one-sixth (*n* = 5) of them had homomeric current such as the figure above (Figure 8). They may form an octamer with the part of TRPC5 from the TRPC1–5 concatemer facing the pore part. In this case, the channel would show the homomeric I–V curve. The other possible structure may be three TRPC5 and one TRPC1 (Kollewe et al., 2022) or one TRPC1 and one TRPC5 with two TRPC4 at the pore region, although the I–V curve was not shown enough to confirm the possibilities. It needs the TRPC1–TRPC5–TRPC5–TRPC5 or TRPC1–TRPC5–TRPC4–TRPC4 concatemer.

Co-expression of TRPC1–5 with Gɑ_i2_(Q205L) significantly enhanced the outwardly rectifying current, especially for stable cell line no. 3 (Figure 4). The observed increase in both cesium and Englerin A currents suggests that heteromeric concatemers exhibit less pore fixation compared to the unstable heteromers used in previous transfections. This indicates an improvement in the channel stability and function with the current concatemer constructs (Kim et al., 2014). However, high-passage cell lines (above 20) of all three of the cell lines no. 3, no. 9, and no. 18 failed to function as heteromers and did not respond to Englerin A treatment. TRPC1–5 Gɑ_q_(Q209L) showed current inhibition in both cesium and Englerin A (Figure 4). The current decreased even when compared to the basal levels.

The fixed stoichiometry of TRPC5–5 and TRPC1–5 can be simply described as the TRPC5 homotetramer and TRPC1–5 1:1 heterotetramer. However, a recent study (Kollewe et al., 2022) confirmed the 1:3 structure of three TRPC4 or TRPC5 and one TRPC1. The studies we have done in this research could be explained as the TRPC1–5 stable cell line could have been generated “with” the endogenous TRPC5s when fixed in the middle of the formation. This may have led the TRPC1–5 heteromer stable cell line to show the heteromeric I–V curve when activated by Englerin A. Transient transfected TRPC1–5 was activated every 10∼15 whole-cell patch-clamp experiments, suggesting that the TRPC1–5 heteromer primarily forms in a 1:1 ratio rather than the more common 3:1 ratio. The fixed 1:1 ratio of TRPC1–5 did not exhibit activation by carbachol, as seen in co-expressions of TRPC5 and TRPC1. This supports the notion that Englerin A is a potent activator, capable of opening TRPC channels under conditions where other activators like riluzole, GSSG, PIP_2_, and Gα are not strong enough to open the channels.

As in the past, FuGENE 6 was considered to be the novel transfection agent to TRPC channels (Myeong et al., 2018; Hong et al., 2015a; Jeon et al., 2012; Ko et al., 2019b), but when transfecting concatemer from the David Beech group, no heteromeric characteristics were observed even when fluorescence protein was clearly seen. We tried cells with and without fluorescence protein for a while, but we could not figure out why the transfection process did not occur. We tried the TurboFect transfection agent that is known to be used for harder transfecting DNA (Lin et al., 2019; Woo et al., 2019; Jeon et al., 2022). Compulsively, DNA was fully functioning when transfected with TurboFect. The David Beech group also used FuGENE HD, which is slightly different from FuGENE 6 (Muraki et al., 2017; Ludlow et al., 2017; Zeng et al., 204; Akbulut et al., 2015; Naylor et al., 2016; Rubaiy et al., 2017a). This confirmed that slight differences in the usage of the transfection agents may lead to large differences when handling structurally different DNA than the previously used ones.

In conclusion, our investigations elucidate that TRPC1–5 heteromeric concatemers are specifically activated by Englerin A as well as active Gα_i_ protein. Also, response to carbachol was observed with the presence of internal GTPγS. Conversely, TRPC5–TRPC5 homomeric concatemers demonstrate responsiveness to both carbachol and Englerin A. This study significantly advances our understanding by indicating that a fixed stoichiometry of 1:1 for TRPC5–TRPC1 concatemers does not optimally enhance their electrophysiological traits. It suggests that alternative stoichiometry, such as one TRPC1 with three TRPC5 or one TRPC1 with one TRPC5 and two TRPC4, might accurately reflect their natural functional assembly (Figure 7) (Kollewe et al., 2022). Consequently, further investigation into diverse stoichiometries is imperative to determine the most efficacious configuration for maximizing the functional properties of TRPC1–5 heteromers. This research not only reconfirms the intricate dynamics between TRPC5 homomers and heteromers but also paves new pathways for exploring the precise molecular configurations that modulate their activity within cellular signaling mechanisms (Kollewe et al., 2022).

## Data Availability

The original contributions presented in the study are included in the article/supplementary material; further inquiries can be directed to the corresponding authors.
